# The Freedom to Pursue Happiness: Belief in Free Will Predicts Life Satisfaction and Positive Affect among Chinese Adolescents

**DOI:** 10.3389/fpsyg.2016.02027

**Published:** 2017-01-04

**Authors:** Chunkai Li, Song Wang, Yajun Zhao, Feng Kong, Jingguang Li

**Affiliations:** ^1^School of Psychology and Cognitive Science, East China Normal UniversityShanghai, China; ^2^Department of Radiology, Huaxi Magnetic Resonance Research Center, West China Hospital of Sichuan UniversityChengdu, China; ^3^College of Sociology and Psychology, Southwest University for NationalitiesChengdu, China; ^4^School of Psychology, Shaanxi Normal UniversityXi’an, China; ^5^College of Education, Dali UniversityDali, China

**Keywords:** belief in free will, subjective well-being, life satisfaction, positive affect, Chinese, adolescents

## Abstract

A small amount of research has examined the association between the belief in free will and subjective well-being (SWB) among Western laypersons from individualist cultures. However, no study has examined this association among participants from collectivist cultures (e.g., Eastern Asian cultures). Therefore, in this study, we explored this association among two large, independent cohorts of Chinese adolescents (*N*_1_ = 1,660; *N*_2_ = 639; high school students). The belief in free will was measured by a self-reported questionnaire (Cohorts 1 and 2) and a two-alternative forced choice question regarding the existence of free will (Cohort 2). SWB included cognitive well-being (life satisfaction) and affective well-being (positive and negative affect) in both cohorts. Data analyses indicated that a stronger belief in free will was consistently associated with higher life satisfaction and positive affect in both cohorts. Our investigation provides evidence supporting the cultural generality of the positive effects of believing in free will on SWB.

## Introduction

The existence of free will has been the subject of debate for centuries among scholars (Libet et al., 1999; Kane, 2005; Baer et al., 2008). Meanwhile, laypersons also have different opinions about free will (Paulhus and Carey, 2011). The present study seeks not to find the ultimate answer in the debate on free will but to explore the practical consequences of individual differences in the belief in free will among laypersons.

In the past decade, psychology has begun to demonstrate the effect of laypersons’ belief in free will on their behavior using correlational or/and experimental methods (for a review, see Baumeister and Monroe, 2014). In correlational investigations, researchers have often used self-reported questionnaires to measure laypersons’ belief in free will (e.g., the FAD-Plus; Paulhus and Carey, 2011) and then correlate it with other measures. These studies have found that a stronger belief in free will is associated with better work performance (Stillman et al., 2010), better academic achievement (Feldman et al., 2016), less conformity (Alquist et al., 2013), and less cheating behavior (Vohs and Schooler, 2008). In experimental investigations, researchers often ask participants to read texts that encourage disbelief in free will and then observe the changes in participants’ behavior. These studies have found that participants induced to disbelieve in free will are more likely to cheat (Vohs and Schooler, 2008), help less and have higher aggression (Baumeister et al., 2009), have less self-control (Rigoni et al., 2012), and present higher conformity (Alquist et al., 2013). In summary, the belief in free will generally predicts positive outcomes and personality traits (Crescioni et al., 2016).

An important research direction regarding the practical consequences of the belief in free will is to investigate the relationship between this belief and happiness. Happiness, also referred to as subjective well-being (SWB) in academic psychology, is necessary for good individual lives and a good society, and research on it is fundamentally essential to behavioral sciences (Diener et al., 2003). If simply believing in free will could help people pursue happiness and be happier, psychologists may develop training programs to increase SWB by facilitating laypersons’ belief in free will. Theoretically, believing in free will means believing that people can freely act to accomplish personal goals and improve life quality. This belief may have benefits on SWB in two aspects. First, the belief in free will may cause an individual to have an increased level of perceived autonomy, which further yielded enhanced SWB (Ryan and Deci, 2000). Second, previous studies suggested that the belief in free will may make an individual more willing to exert self-control (Rigoni et al., 2012) and perform better (Stillman et al., 2010; Feldman et al., 2016). For example, if an individual believes that s/he can freely act to achieve her/his desire, there might be more incentive for self-control and deliberate effort, which further lead to success. Given that both self-control and success are vital predictors of the SWB (Lyubomirsky et al., 2005; Hofmann et al., 2014), the belief in free will may associated with enhanced SWB.

Until now, only three empirical studies have reported the association between the belief in free will and SWB. Two of these studies indicated that the belief in free will was positively correlated with life satisfaction among college students (*r* = 0.17, *N* = 234; Bergner and Ramon, 2013) and adult employees (*r* = 0.32, *N* = 65; Stillman et al., 2010), although testing this association was not the main purpose of their studies. The third study purposefully tested this association (Crescioni et al., 2016). The study indicated that the belief in free will was positively correlated with global subjective happiness (*r* = 0.56) and life satisfaction (*r* = 0.59) among adults (*N* = 44). Additionally, regression analyses conducted on another independent sample of adults (*N* = 77) indicated that the belief in free will still predicted life satisfaction, even after controlling for two other agency-related variables (i.e., locus of control and implicit theory). Altogether, these studies provide valuable evidence to support the link between the belief in free will and SWB.

However, all three of the abovementioned studies that tested the relationship between the belief in free will and SWB were conducted with US participants (Stillman et al., 2010; Bergner and Ramon, 2013; Crescioni et al., 2016). Therefore, it is worth examining whether this effect is fundamental to human nature or specific to contemporary Western culture. Prior research has revealed that individuals from the West and those from Eastern Asia have some different core ideas and conceptualizations of free will. Specifically, compared with Eastern Asians, Westerners attribute more value to individual freedom (vs. society rule), personal choice (vs. group goals), and independence (vs. interdependence) (Markus and Kitayama, 1991; Kashima et al., 1995; Nisbett, 2003). Accordingly, the behavioral outcomes of the belief in free will might be also different across cultures. Therefore, it is intriguing to ask whether the laypersons’ belief in free will has a positive influence on SWB among individuals from collectivistic cultures, such as China.

In the investigation reported here, we examined the association between the belief in free will and SWB in two studies. In Study 1, we used a self-reported questionnaire to measure the belief in free will and to correlate it with cognitive well-being (life satisfaction) and affective well-being (positive and negative affect). In Study 2, we used a two-alternative forced choice philosophical question regarding the existence of free will to directly classify the participants into two categories: free will believers and determinism believers. Then, we compared their scores on the aforementioned SWB measures.

## Study 1

The main goal of Study 1 was to perform an initial test of the hypothesis that the belief in free will is associated with SWB by assessing its association with life satisfaction and positive and negative affect.

Moreover, if the belief in free will and one or all indicators of SWB were correlated, we would further test whether these associations held when the Big Five personality traits (Costa and McCrae, 1992) were controlled. It is well established that Big Five personality traits are powerful predictors of SWB. For instance, a meta-analysis revealed that all of the Big Five personality traits are correlated with SWB (*r*: 0.11 ∼ 0.22) (DeNeve and Cooper, 1998). Another meta-analysis estimated that the Big Five personality traits may account for as much as 39% (or 63%, disattenuated of measurement error) of SWB variance (Steel et al., 2008). Two studies reported associations between the belief in free will and Big Five personality traits (Stillman et al., 2010; Paulhus and Carey, 2011). Specifically, Paulhus and Carey (2011) found that the belief in free will, measured by the FAD-Plus, was positively correlated with extraversion and agreeableness, and Stillman et al. (2010) reported that the belief in free will, measured by the FAD (a previous version of the FAD-Plus), was positively correlated with the other three Big Five facets, i.e., openness, conscientiousness, and emotional stability. Taken together, the Big Five personality traits simultaneously correlate with the belief in free will and SWB. Thus, if we observe the association between the belief in free will and SWB, we cannot exclude a simple possibility: the measure of the belief in free will itself contains items overlapping with Big Five personality traits. Therefore, to strictly examine the association between the belief in free will and SWB, it is necessary to control for the Big Five personality traits.

### Material and Methods

#### Participants and Procedure

The participants were 1,660 10th-grade students recruited from three high schools in Chengdu, China (53.5% females; mean age = 16.3 years, *SD* = 0.56 years). The Medical Ethics Committee of Dali University approved the study. We obtained written consent from all participants and their parents. Participants completed a battery of paper-based personality questionnaires, including the FAD-Plus, the Satisfaction with Life Scale (SWLS), the Positive and Negative Affect Schedule (PANAS), and the NEO-FFI. Participants completed the battery of questionnaires in their respective classrooms.

#### Measures

##### The Free will and Determinism Plus

The FAD-Plus is a 27-item Likert scale that measures beliefs in free will and related constructs (Paulhus and Carey, 2011). The Chinese version of the FAD-Plus was first translated by the first author of the current manuscript; then, the last author back-translated it into English. Discrepancies between the Chinese and English versions were identified and discussed within a group that involved the first author, the last author, and a native English speaker (all of whom are fluent in Chinese) until they reached a consensus regarding the Chinese version of the FAD-Plus. For the purpose of the current study, we used only the Free Will subscale, containing seven items. Example items include “People have complete control over the decisions they make” and “Criminals are totally responsible for the bad things they do.” In the current study, Cronbach’s α for this subscale was 0.71, which was comparable to those in the original scale (α = 0.69/0.70; Paulhus and Carey, 2011).

##### Satisfaction with Life Scale (SWLS)

The SWLS is a 5-item questionnaire that contains only one dimension measuring global cognitive SWB (Diener et al., 1985). Participants respond to each item using a 7-point Likert scale, with response options ranging from 1 (strongly disagree) to 7 (strongly agree). Example items include “In most ways my life is close to my ideal” and “I am satisfied with my life.” The Chinese version of the SWLS demonstrates satisfactory psychometric properties with regard to Chinese adolescents (Sun and Shek, 2010). In the current study, Cronbach’s α for the SWLS was 0.77.

##### Positive and Negative Affect Schedule (PANAS)

The PANAS is a widely used tool to measure general affect (Watson et al., 1988). The scale includes two 10-items subscales, positive affect (PA) and negative affect (NA). Participants were instructed to indicate how often they have felt a particular affect during the past month on a 5-point Likert scale, ranging from 1 (very slightly or not at all) to 5 (extremely). The PANAS was translated into Chinese in the same way the FAD-Plus translation was performed, as described above. In the current study, Cronbach’s α for PA and NA were 0.88 and 0.86, respectively.

##### NEO-FFI

The NEO-FFI is a self-report instrument measuring the Big Five personality traits. This inventory includes 60 items rated on a 5-point Likert-type scale ranging from 1 (strongly disagree) to 5 (strongly agree). Example items include “I am a very active person” and “I am seldom sad or depressed.” The NEO-FFI was translated by Yang et al. (1999). The Chinese version of the NEO-FFI demonstrates satisfactory validity with regard to Chinese undergraduates (Yao and Liang, 2010) and adolescent students (Chen and Zhang, 2011). In the current study, Cronbach’s α coefficients for the NEO-FFI subscales were 0.74, 0.79, 0.56, 0.60, and 0.75 for Neuroticism, Extraversion, Openness, Agreeableness, and Conscientiousness, respectively. Notably, Cronbach’s α coefficients for Openness and Agreeableness were lower than those reported in the original NEO-FFI (αs > 0.75). These lower coefficients (i.e., less than 0.70) were consistent with those obtained in some other studies conducted among Chinese participants (Zhang, 2003; Yao and Liang, 2010). These low reliability coefficients may reflect that Openness and Agreeableness have different meanings in Chinese culture or reflect an inaccurate translation of the English scales (Zhang, 2003). However, to perform a comprehensive investigation across all Big Five domains, the data from these two subscales were still included in subsequent analyses.

### Results and Discussion

The descriptive statistics (i.e., means and standard deviations) and the correlations between measures are presented in **Table 1**. Critically, the belief in free will was positively correlated with life satisfaction and PA but negatively correlated with NA.

**Table 1:** Descriptive statistics and correlations in Study 1.

	*M*	*SD*	BFW	LS	PA	NA	O	C	E	A	N
BFW	4.41	0.78	–								
LS	4.05	1.01	0.19^∗∗^	–							
PA	3.27	0.66	0.22^∗∗^	0.33^∗∗^	–						
NA	2.55	0.65	-0.13^∗∗^	-0.22^∗∗^	-0.23^∗∗^	–					
O	2.99	0.58	0.17^∗∗^	0.17^∗∗^	0.38^∗∗^	-0.16^∗∗^	–				
C	3.45	0.51	0.17^∗∗^	0.28^∗∗^	0.44^∗∗^	-0.31^∗∗^	0.37^∗∗^	–			
E	3.35	0.40	0.21^∗∗^	0.28^∗∗^	0.52^∗∗^	-0.25^∗∗^	0.26^∗∗^	0.27^∗∗^	–		
A	3.43	0.40	0.18^∗∗^	0.27^∗∗^	0.16^∗∗^	-0.34^∗∗^	0.24^∗∗^	0.31^∗∗^	0.24^∗∗^	–	
N	3.19	0.44	-0.12^∗∗^	-0.29^∗∗^	-0.35^∗∗^	0.61^∗∗^	-0.14^∗∗^	-0.37^∗∗^	-0.38^∗∗^	-0.30^∗∗^	–

*BFW, belief in free will; LS, life satisfaction; PA, positive affect; NA, negative affect; O, openness; C, conscientiousness; E, extraversion; A, agreeableness; N, neuroticism. ^∗∗^*p* < 0.01.*

Moreover, the Big Five personality traits were correlated with three indicators of SWB. Therefore, we performed three hierarchical regression analyses to examine whether the belief in free will explained additional variance in SWB beyond the variance explained by the Big Five personality traits. In all regression analyses, Big Five personality traits were entered first; then, the belief in free will was entered. Life satisfaction, PA, and NA were dependent variables for each regression analysis. **Table 2** presents the outcomes of these regression analyses. Importantly, when predicting life satisfaction and PA, belief in free will explained additional variance beyond other predictors (i.e., 1% and 1%, *p*s < 0.001). However, the belief in free will no longer contributed significantly to NA (*p* = 0.31).

**Table 2:** Hierarchical regression models for subjective well-being (SWB) in Study 1.

	Life satisfaction			Positive affect				Negative affect		
	*B* (*SE*)	β	Δ*R*^2^	*B* (*SE*)	β	Δ*R*^2^	*B* (*SE*)	β	Δ*R*^2^
Step 1			0.16^∗∗∗^			0.41^∗∗∗^			0.41^∗∗∗^
Neuroticism	-0.24 (0.05)	-0.14		-0.14 (0.03)	-0.13		0.61 (0.03)	0.55	
Extraversion	0.27 (0.05)	0.14		0.48 (0.03)	0.37		0.02 (0.03)	0.02	
Openness	0.08 (0.07)	0.03		0.34 (0.04)	0.21		-0.04 (0.04)	-0.02	
Agreeableness	0.37 (0.07)	0.15		-0.15 (0.04)	-0.09		-0.27 (0.04)	-0.17	
Conscientiousness	0.29 (0.06)	0.13		0.36 (0.04)	0.24		-0.08 (0.04)	-0.06	
Step 2			0.01^∗∗∗^			0.01^∗∗∗^			<0.001
Neuroticism	-0.24 (0.05)	-0.14		-0.14 (0.03)	-0.12		0.61 (0.03)	0.55	
Extraversion	0.24 (0.05)	0.12		0.46 (0.03)	0.36		0.03 (0.03)	0.02	
Openness	0.06 (0.07)	0.02		0.33 (0.04)	0.20		-0.03 (0.04)	-0.02	
Agreeableness	0.35 (0.07)	0.14		-0.16 (0.04)	-0.10		-0.26 (0.04)	-0.16	
Conscientiousness	0.27 (0.06)	0.12		0.35 (0.04)	0.24		-0.08 (0.04)	-0.05	
Belief in Free Will	0.14 (0.03)	0.10		0.06 (0.02)	0.07		-0.02 (0.02)	-0.02	

*^∗∗∗^*p* < 0.001.*

In summary, Study 1 suggested that the belief in free will is associated with better life satisfaction and higher PA; its predictive power extends even beyond major dimensions of personality (i.e., Big Five personality traits). Next, we proceeded to Study 2 to replicate these results.

## Study 2

One important limitation of Study 1 is that the FAD-Plus may measure psychological traits other than the belief in free will, and these traits may also correlate with the SWB. For example, one FAD-Plus item (i.e., “Criminals are totally responsible for the bad things they do”) seemingly overlaps with moral responsibility. Another FAD-Plus item (i.e., “Strength of mind can always overcome the body’s desires”) seemingly reflects the self-control. Thus, although Study 1 ruled out the influence of the Big Five personality traits, the observed association between the belief in free will and SWB may still due to the “unpurified” nature of the FAD-Plus.

Therefore, it is necessary to measure the belief in free will in a direct and pure way that excludes any possibly confounding factors. To fulfill this goal, Study 2 followed the method developed by Nichols and Knobe (2007) to measure the belief in free will. That is, we used one two-alternative forced choice philosophical question regarding the existence of free will to directly classify the participants into two categories: free will believers and determinism believers. Importantly, this question did not use abstract philosophical terms but created concrete scenarios to facilitate laypersons’ understanding. Then, we directly compare the SWB levels of free will believers and determinism believers.

### Material and Methods

#### Participants and Procedure

The participants were 639 10th-grade high school students recruited from one high school in Chengdu, China (58.4% females; mean age = 16.2 years, *SD* = 0.55 years). The Medical Ethics Committee of Dali University approved the study. We obtained written consent from all participants and their parents. Participants completed a battery of paper-based tests, including a two-alternative forced choice philosophical question regarding the existence of free will, the FAD-Plus, the SWLS, and the PANAS. Participants completed the battery of questionnaires in their respective classrooms.

#### Measures

The FAD-Plus, the SWLS, and the PANAS were the same as in Study 1. We calculated the reliability scores for the current dataset. Cronbach’s α for the belief in free will subscale in the FAD-Plus was 0.64. Cronbach’s α for life satisfaction in the SWLS was 0.80. Cronbach’s αs for PA and NA in the PANAS were 0.89 and 0.85, respectively. All these reliability scores were comparable to those in Study 1.

The single philosophical question to measure the belief in free will was designed by Nichols and Knobe (2007) and has been successfully used in Chinese undergraduate students (Sarkissian et al., 2010). Here, the test materials were translated into Chinese from those used by Nichols and Knobe (2007). Participants first read descriptions of a deterministic scenario and a free will scenario.

Imagine a universe (Universe A) in which everything that happens is completely caused by whatever happened before it. This is true from the very beginning of the universe, so what happened in the beginning of the universe caused what happened next, and so on right up until the present. For example one day. John decided to have French fries at lunch. Like everything else, this decision was completely caused by what happened before it. So, if everything in this universe was exactly the same up until John made his decision, then it had to happen that John would decide to have French fries.

Now imagine a universe (Universe B) in which almost everything that happens is completely caused by whatever happened before it. The one exception is human decision making. For example, one day Mary decided to have French fries at lunch. Since a person’s decision in this universe is not completely caused by what happened before it, even if everything in the universe was exactly the same up until Mary made her decision, it did not have to happen that Mary would decide to have French fries. She could have decided to have something different.

Then, participants were required to make a two-alternative forced choice: “Which of these universes do you think is most like ours?” Participants who chose Universe A were labeled as determinism believers, and the rest were labeled as free will believers.

### Results and Discussion

First, consistent with previous studies (Nichols and Knobe, 2007; Sarkissian, et al., 2010), a majority of participants (*N* = 546, 85.4%) chose Universe B (i.e., the free will scenario). Then, we compared the scores on the free will subscale of the FAD-Plus between free will believers and determinism believers. Indeed, compared with determinism believers, free will believers scored significantly higher, *t*(637) = 2.50, *p* < 0.01, Cohen’s *d* = 0.28 (**Table 3**). Therefore, these results provided evidence for convergent validity regarding two different measures of the belief in free will.

**Table 3:** Mean scores and standard deviations (SD) of SWB indicators among free will believers and determinism believers in Study 2.

	Determinism believers (*N* = 93)	Free will believers (*N* = 546)
Free will score of FAD-Plus	3.29 (0.54)	3.44 (0.55)
Life satisfaction	3.99 (1.14)	4.21 (1.02)
Positive affect	3.22 (0.64)	3.37 (0.63)
Negative affect	2.67 (0.66)	2.46 (0.57)

Next, we correlated the scores on the free will subscale of the FAD-Plus with all three indicators of SWB, aiming to replicate part of the findings in Study 1. Indeed, similar patterns of correlations were found. That is, the belief in free will measured by the FAD-Plus was positively correlated with life satisfaction (*r* = 0.22, *p* < 0.001) and PA (*r* = 0.22, *p* < 0.001) but was negatively correlated with NA (*r* = -0.08, *p* = 0.047).

Finally, and most critically, we compared the SWB level between free will believers and determinism believers (**Table 3**). Compared with determinism believers, free will believers scored higher in life satisfaction [*t*(637) = 1.89, *p* = 0.06, Cohen’s *d* = 0.20] and PA [*t*(637) = 2.12, *p* = 0.03, Cohen’s *d* = 0.24], and lower in NA [*t*(637) = 3.28, *p* < 0.001, Cohen’s *d* = 0.34].

## General Discussion

In this investigation, we explored the association between the belief in free will and SWB, including cognitive well-being (life satisfaction) and affective well-being (positive and negative affect), among two large independent cohorts of Chinese adolescents in high school. In Study 1, the belief in free will, measured by the FAD-Plus, was associated with higher life satisfaction and PA, controlling for the Big Five personality traits. In Study 2, we directly classified participants as free will believers (85%) and determinism believers (15%) based on a single philosophical question regarding the existence of free will. We found that free will believers had higher life satisfaction and PA, but lower NA than determinism believers. Admittedly, the association pattern between the belief in free will and SWB was not totally consistent across Studies 1 and 2, which may have been caused by different measures of the belief in free will and other methodological issues (e.g., different participant groups or sampling errors). However, both studies consistently revealed that the belief in free will was associated with higher life satisfaction and PA among Chinese adolescents.

One critical and novel finding of the current study is that the belief in free will still predicts SWB (at least life satisfaction and PA) in the Chinese culture. The Chinese culture is often labeled as a collectivistic culture that does not emphasize the concepts related to the belief in free will, such as freedom, personal choice, and independence (Markus and Kitayama, 1991; Kashima et al., 1995). Moreover, unlike Western philosophy, traditional Chinese philosophy seldom directly studies the concept of free will (Feng, 1990). For instance, there is almost no mention of free will or freedom in one of the famous classic describing the history of Chinese philosophy, *A History of Chinese Philosophy* by the widely recognized Chinese Philosophers Feng (1983). However, consistent with a previous study conducted on Chinese undergraduate students (Sarkissian et al., 2010), Study 2 of the current investigation suggested that most Chinese adolescents do believe in free will. Moreover, our study revealed that this belief still has a positive effect on SWB in Chinese and implied the possibility of the cultural generality of the effect. This evidence further implies that the belief in free will and its benefit might be a universal human nature. Future studies could test these hypotheses with different cultures.

Moreover, the methodology of our study differs from previous investigations in three other distinct ways. First, compared with previous investigations, we used relatively comprehensive measures to index the belief in free will. It is worth mentioning that no previous studies have directly compared the SWB of free will and determinism believers, which was performed in Study 2 of the current investigation. Therefore, this result provides converging evidence to support the previous observation of the association between the belief in free will measured by the FAD-Plus and SWB (Stillman et al., 2010; Bergner and Ramon, 2013; Crescioni et al., 2016).

Second, compared with previous investigations, we used relatively comprehensive measures to index SWB (Diener et al., 2003), including both cognitive (life satisfaction) and affective well-being (positive and negative affect) simultaneously. As Diener et al. (1999) recommended, major components of SWB should be assessed separately, and this could help in understanding the different relationships among predictor variables and the components of SWB. Interestingly, we indeed found that the belief in free will had different influences on these different indices in both Study 1 and Study 2. These results are not surprising because it is known that these indicators are relatively independent components (e.g., Watson et al., 1988). However, future studies are needed to explain the mechanisms underlying these dissociations.

Third, the present investigation extended the previous findings from the adult population to the adolescent population. Although the philosophical concept of free will might be difficult for adolescents to understand, they may also possess opinions about free will, at least on the layperson level. For example, high school students may have different understandings of and attitudes towards the statement “People have complete control over the decisions they make,” an item designed to measure laypersons’ belief in free will from the FAD-Plus (Paulhus and Carey, 2011), and they could also understand the differences between two concrete scenarios in Study 2. Thus, our study invites a broader investigation of whether adolescents’ beliefs in free will have similar behavioral consequences as adults’ beliefs in free will, such as academic performance (Feldman et al., 2016), moral behavior (Vohs and Schooler, 2008), and self-control (Rigoni et al., 2012).

Finally, the current investigation has one major limitation that deserves consideration. That is, we did not provide evidence^1^ that can support the causal relationship between the belief in free will and SWB. Therefore, we cannot rule out the possibility that SWB causes an increased belief in free will. Future investigations may consider using experimental or longitudinal designs to explore the causal direction in this association.

## Conclusion

Free will is not only an abstract concept for philosophers; it also plays a vital role in laypersons’ daily life. In the present study, we found that a stronger belief in free will was associated with increased life satisfaction and PA in two independent large samples of Chinese adolescents. Our investigation provides evidence supporting the cultural generality of the positive effects of believing in free will on SWB.

## Ethics Statement

The Medical Ethics Committee of Dali University approved the study. Prior to testing, we obtained written consent from all participants and their parents.

## Author Contributions

JL conceived of and designed the study; SW, YZ, FK, and JL contributed to data collection; CL, SW, and JL analyzed data and wrote the paper. All authors reviewed and approved the manuscript.

## Conflict of Interest Statement

The authors declare that the research was conducted in the absence of any commercial or financial relationships that could be construed as a potential conflict of interest.

## Footnotes

1 To further investigate the causal relationship between the belief in free will and SWB, we conducted an additional experiment. Although we did not find any conclusive results, we still summarized this experiment here for the reference of future studies. Specifically, we tested the hypothesis that inducing disbelief in free will would make people less happy. Followed the paradigm developed by Vohs and Schooler (2008) and widely used by other researchers (e.g., Shariff et al., 2014; Crescioni et al., 2016), we experimentally manipulated participants’ beliefs in free will by asking them to read materials that reject the idea of free will. Participants were 131 Chinese undergraduate students who participated in receiving credit in their psychology course. First, they were randomly assigned to read one of two passages from *The Astonishing Hypothesis*, written by the Nobel-prize-winning biologist Francis Crick (1994). These materials were used in Vohs and Schooler (2008)’s study and were translated from English to Chinese by the authors of the current manuscript. In the *anti-free-will condition* (*N* = 65, 56 females), the passage rejects the idea of free will and advocates a view of scientific determinism. In the control condition (*N* = 66, 55 females), the passage describes the mechanism and research history of consciousness. Next, participants completed the free will subscale of the FAD plus (Cronbach’s α = 0.63). Finally, participants completed two questionnaires to measure their cognitive and affective well-being, respectively. As in Study 1 and 2, the cognitive well-being was indexed by the SWLS (Cronbach’s α = 0.72). The affective well-being was indexed by the Faces Scale, a 7-point non-verbal scale consisting of seven emotional face icons (Andrews and Crandall, 1976). Each face icon consisted of a circle with two unchanged eyes and a mouth which varied from a smile to a frown across seven levels. Participants were asked to choose a face icon that could best represent their current feelings. It is worth mentioning that we did not use the PANAS to measure the affective well-being, given that several studies have already demonstrated that the manipulation of the disbelieving in free will did not influence the scores of the PANAS (e.g., Vohs and Schooler, 2008; Rigoni et al., 2012; Shariff et al., 2014). Participants in the *anti-free-will condition* (*M* = 3.88, *SD* = 0.68) reported less beliefs in free will than participants in the control condition (*M* = 4.01, *SD* = 0.69). Although the direction of group difference was in accordance with the direction of manipulation (i.e., reading the anti-free-will passage reduces the belief in free will), this effect was not statistically significant [*t*(129) = 1.21, *p* = 0.27, Cohen’s *d* = 0.20]. Therefore, the experimental manipulation might only have a limited effect on altering participants’ beliefs. To test our hypothesis that discouraging a belief in free will would reduce happiness level, we compared the scores of SWB measures across groups. There was almost no group difference in life satisfaction, *t* < 1, *p* = 0.80. Moreover, participants in the *anti-free-will condition* (*M* = 5.40, *SD* = 0.11) reported less affective well-being than participants in the control condition (*M* = 5.68, *SD* = 0.10). The direction of the effect was in accordance with the hypothesis, but this effect was still not statistically significant [*t*(129) = 1.50, *p* = 0.16, Cohen’s *d* = 0.26]. In summary, the aforementioned preliminary experiment did not provide evidence to support the hypothesis that discouraging a belief in free will would lead to a change in happiness. The reasons for these null results are complex. First, it is possible that believing in free will could enhance SWB, but it may take much longer time (e.g., several days or months). Second, the priming materials (i.e., *The Astonishing Hypothesis*) might be difficult to be understood by participants in the current experiment, which may further reduce the effect of experimental manipulation. Third, the current sample size might be not enough to detect a real experimental effect. Nevertheless, to fully understand the causal relationship between the belief in free will and SWB, future experimental studies with improved methodologies are needed.
